# Reversible modulation of SIRT1 activity in a mouse strain

**DOI:** 10.1371/journal.pone.0173002

**Published:** 2017-03-08

**Authors:** Katherine V. Clark-Knowles, Xiaohong He, Karen Jardine, Josée Coulombe, Danielle Dewar-Darch, Annabelle Z. Caron, Douglas A. Gray, Michael W. McBurney

**Affiliations:** 1 Program in Cancer Therapeutics, Ottawa Hospital Research Institute, Ottawa, Canada; 2 Department of Biochemistry, Microbiology and Immunology, University of Ottawa, Ottawa, Canada; 3 Department of Medicine, University of Ottawa, Ottawa, Canada; Universite de Geneve, SWITZERLAND

## Abstract

The SIRT1 protein deacetylase is reported to have a remarkably wide spectrum of biological functions affecting such varied processes as aging, cancer, metabolism, neurodegeneration and immunity. However, the SIRT1 literature is also full of contradictions. To help establish the role(s) of SIRT1 in these and other biological processes, we set out to create a mouse in which the SIRT1 activity could be toggled between on and off states by fusing the estrogen receptor ligand-binding domain (ER) to the C terminus of the SIRT1 protein. We found that the catalytic activity of the SIRT1-ER fusion protein increased 4–5 fold in cells treated with its ligand, 4-hydroxy-tamoxifen (4OHT). The 4OHT-induced activation of SIRT1-ER was due in large part to a 2 to 4-fold increase in abundance of the SIRT1-ER protein in cells in culture and in tissues *in vivo*. This increase is reversible and is a consequence of 4OHT-induced stabilization of the SIRT1-ER protein. Since changes in SIRT1 level or activity of 2–4 fold are frequently reported to be sufficient to affect its biological functions, this mouse should be helpful in establishing the causal relationships between SIRT1 and the diseases and processes it affects.

## Introduction

The proteins encoded by the *sir2* genes in yeast, *C*. *elegans*, *D*. *melanogaster*, and mouse have been reported to prolong lifespan and increase organismal resistance to stress [1–4]. Like other members of the sir2 family, the mammalian SIRT1 protein contains a catalytic domain that couples NAD^+^ hydrolysis to protein deacetylation [5–7]. The number of documented acetylated protein substrates of SIRT1 now exceeds 100 [8], and there is evidence that the SIRT1 protein has functions not dependent on its catalytic activity [9–15].

SIRT1 is a nuclear protein expressed in all tissues albeit at variable levels [16]. Knockouts of the *sirt1* gene have been created [17, 18] and animals lacking SIRT1 are viable on outbred strains.

SIRT1 has been reported to have effects on a wide variety of cellular functions yet there is a remarkable amount of controversy because many of the reports are contradictory. This is particularly true for the role of SIRT1 in cancer [8]. SIRT1 is reported to be both a tumor suppressor and an oncogene (reviewed in [19]) while other reports indicate no effect of SIRT1 on tumorigenesis [20, 21]. To establish the roles of other oncogenic or tumor suppressor proteins in tumorigenesis, reversible activation of protein function can be a valuable tool [22]. One way that protein function can be reversibly toggled between active and inactive states is by fusion of the test protein to the hormone binding domain of a steroid hormone receptor [23] [24]. Particularly useful in mammalian systems is the estrogen receptor that has been modified to bind 4-hydroxytamoxifen (4OHT) [25]. We set out to create a mouse strain in which the endogenous *sirt1* gene is modified to encode a full length SIRT1 protein fused in frame to the hormone binding region of the mouse estrogen receptor alpha (ERα) modified to bind to 4OHT [25]. We report below the characteristics of a strain of mice that expresses a SIRT1-ER fusion protein from the endogenous *sirt1* locus. The abundance and activity of SIRT1-ER is reversibly augmented with 4OHT *in vitro* and by tamoxifen *in vivo*.

## Materials and methods

### Animal experiments

All animal experimentation was carried out in accordance with the guidelines of the Canadian Council for Animal Care with protocols approved by the Animal Care Committee of the University of Ottawa. Animals were maintained in a pathogen-free facility at 24°C with a 12-h light-dark cycle.

The mouse line carrying the modified *sirt1*^*ER*^ allele was created by aggregating *sirt1*^*+/ER*^ embryonic stem cells with morulae of CD1 strain mice, culturing them to the blastocyst stage and transferring them to the uteri of pseudopregnant CD1 females as previously described [26]. Chimeric animals were identified by coat pigmentation. Male chimeras were mated to strain 129/Sv females and offspring inheriting the *sirt1*^*ER*^ allele were identified at weaning by taking a small tail or ear clip to isolate DNA. The *sirt1*^*+*^ and *sirt1*^*ER*^ alleles were identified by PCR amplification of genomic DNA using the primers: 5’-TGATGAACAATGGCAAAGC-3’, and 5’-TGTCAAAGAAACAGCGTTGG-3’. Amplification was carried out as follows: 95°C 3 min., 95°C 15 sec., 57°C 15 sec., 72°C 30 sec., 72°C 60 sec., 4°C ∞ (repeating middle steps 34 times). The *sirt1*^*+*^ allele yields a 185 bp fragment while the *sirt1*^*ER*^ allele fragment is 485 bp.

**Fig 1:**
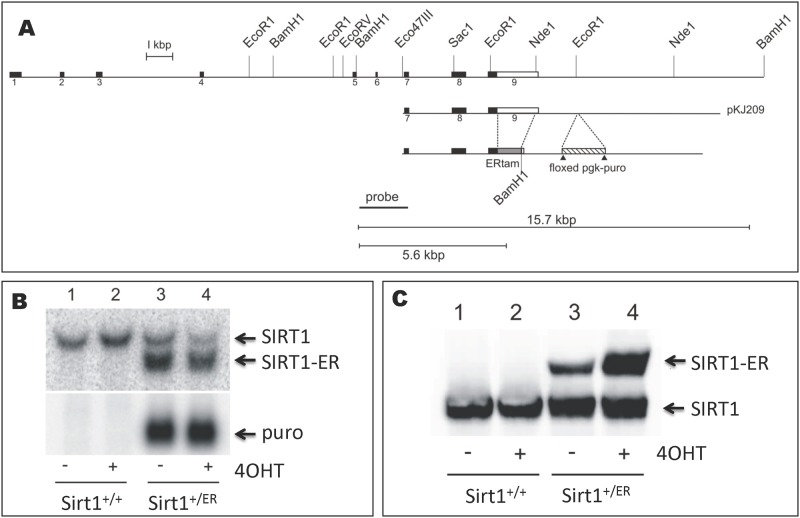
Creation of the *sirt1*^*ER*^ allele by homologous recombination. A. The *sirt1* gene is depicted with its 9 exons indicated as boxes above the line. A replacement vector was constructed from a cloned genomic fragment (pKJ209). The region coding for amino acids 281–599 of the mutated murine ERα [29] was fused in frame downstream of the SIRT1 coding region and all but 130 bp of the 3’UTR of the *sirt1* gene was removed. A selectable *pgk-puro* gene was inserted downstream between 2 *loxP* sites. This insertion vector was electroporated into R1 [28] ES cells and clones carrying the correctly targeted insertion were identified by Southern blots of BamH1 digested cellular DNA probed with the region identified. B. Northern blot of RNA isolated from the parental embryonic stem cells (*sirt1*^*+/+*^) and a subclone carrying the correctly inserted DNA fragment (*sirt1*^*+/ER*^). The blot was probed with the *sirt1* cDNA and the *puro* gene. Bands corresponding to the native SIRT1 mRNA and the mRNA encoding SIRT1-ER are shown in the upper panel. Cells were treated for 24 hr with 30 nM 4-hydroxytamoxifen (4OHT) with little effect on the abundance of the mRNAs. C. Western blot of protein isolated from *sirt1*^*+/+*^ and *sirt1*^*+/ER*^ ES cells after treatment with 4OHT for 24 hr. The locations of the SIRT1 and the SIRT1-ER proteins are shown. The abundance of the SIRT1-ER fusion protein was increased about 3–4 fold following 4OHT treatment.

Female animals carrying the *sirt1*^*ER*^ allele were mated to males carrying the cre recombinase driven by the protamine promoter (JAX stock 003328, 129Tg(prm-cre) 580g/J transgene) and male offspring carrying both the *sirt1*^*ER*^ allele and prm-cre transgene were mated to 129/Sv females. Offspring from these matings had deleted the *pgk-puro* gene that had been inserted downstream of the *sirt1*^*ER*^ allele (Fig 1A). Following 5 backcrosses to strain 129/Sv, *sirt1*^*+/ER*^ animals were intercrossed and homozygous *sirt1*^*ER/ER*^ mice were subsequently maintained by brother-sister crosses.

Animals were normally fed regular chow (Teklad Global 2018, ENVIGO, Madison, WI, USA) or were transferred to chow containing tamoxifen at low (30 mg/kg), intermediate (110 mg/kg) or high dose (500 mg/kg) (Teklad custom rodent diet, ENVIGO). Oral glucose tolerance tests were performed as described [15]

Magnetic resonance imaging of the heads of mice was carried out as described [27]. Measurements were performed using ImageJ software. Images were imported into Adobe Photoshop CS6 software, landmarks identified on coronal sections, false colored and overlayed.

Extraorbital lacrimal and salivary glands were dissected upon necropsy from young (2–6 months) and older (7–12 months) mice. Specimens were fixed in 10% neutral buffered formalin and paraffin embedded. Three separate hematoxylin and eosin stained sections per gland, spaced 20 μm apart, were assessed for the presence of nests of lymphocytes.

### Cell culture experiments

All cells were cultured in Dulbecco’s MEM with high glucose and 10% fetal calf serum. The mouse embryonic stem cell line, R1 [28], was cultured in medium supplemented with LIF and 2-mercaptoethanol as previously described [26] and used as the recipient (by electroporation) of the knock-in vector shown in Fig 1A. HEK293T cells were transfected using jetPRIME (#114–07, #712–60, Polyplus transfection, NY, USA) according to the protocol recommended by the manufacturer.

Mouse embryo fibroblasts (MEFs) were isolated from day 12.5 embryos. Briefly, individual embryos were dissected free of membranes, the heads and internal organs removed, and the carcasses incubated for 10 minutes in 0.25% trypsin and 1.0 mM EDTA in phosphate buffered saline. Following vigorous trituration, the cells were plated in growth medium. These cultures were passaged at 3 or 4-day intervals. After 6 to 10 passages, each culture entered a crisis phase and after an additional 1–2 weeks, immortalized cells emerged. Cultures were used for experiments normally between 20 and 50 passages.

Chemicals added to culture medium included 4-hydroxytamoxifen (4OHT, Sigma, 30–100 nM), geldanamycin (Sigma, 0.5 μg/ml), MG132 (Sigma, 1 μM), actinomycin D (Boehringer, 5 μg/ml), cycloheximide (Sigma, 10 μg/ml). In addition, trichostatin A (TSA, Sigma, 4 μM) and nicotinamide (NAM, Sigma, 10 mM) were added to SIRT-Glo assays.

Protein gel electrophoresis and immunoblots were carried out as described [27]. Protein was extracted from frozen tissues via mechanical homogenization in Tris lysis buffer (20 mM Tris, pH 8.0) or RIPA buffer (25 mM Tris pH 7, 150 mM NaCl, 0.1% SDS, 0.5% sodium deoxycholate, 1% NP-40) followed by a freeze-thaw cycle and centrifugation. Protein (30 μg) was loaded onto gradient gels and transferred to nitrocellulose membranes. Membranes were probed with the following antibodies: polyclonal rabbit antibody to the N-terminal sequence of SIRT1 (#07–131, Millipore, CA, USA), mouse monoclonal antibody to p53 (#2524S, Cell Signaling Technology, MA, USA), rabbit polyclonal to acetyl-p53 (#2570S, Cell Signaling Technology, MA, USA), rabbit polyclonal to mouse ERα (#sc-542, Santa Cruz Biotechnology, Inc. CA, USA). Densitometry analysis was performed using ImageJ software.

Quantitative gene amplification was carried out using an ABI 7500 Fast PCR machine. Briefly, RNA was isolated from cells using the GeneElute Mammalian Total RNA Miniprep Kit (Sigma) and 1 μg of RNA was reverse transcribed using the High Capacity cDNA Reverse Transcription Kit (ABI). cDNA was diluted 1:50 in nuclease free water and 5 μL used per reaction under standard conditions (95°C hold 20 sec followed by 40 cycles at 95°C 3 sec, 60°C 30 sec). Inventoried Taqman Assays were obtained from ABI and reactions run in triplicate to determine the relative levels of SIRT1 mRNA (Mm00490758_m1) and 18S rRNA (Hs99999901_s1).

### Enzyme assay

The SIRT-Glo^™^ assay (Promega, Madison, WI, USA) was used to measure sirtuin activity and luminescence was measured using a Synergy MX plate reader (Biotek, Winooski, VT, USA). Cell lysates were prepared in 20 mM Tris, pH 8.0 and subject to immunoprecipitation with antibody directed to the N-terminus of SIRT1 (EMD Millipore, Etobicoke, ON, Canada). The immunoprecipitates were used to prime sirtuin assays carried out in the presence of TSA (4 μM) or TSA and nicotinamide (10 mM).

## Results

### Creation and expression of the *sirt*^*ER*^ allele

We used homologous recombination in the R1 line of embryonic stem (ES) cells [28] to modify the *sirt1* gene. A knock-in vector (Fig 1A) was constructed that replaced all but the 3’ 130 bases of the 1.8 kb 3’UTR of the SIRT1 transcript with a region encoding the hormone-binding domain of the mouse estrogen receptor alpha (ERα) containing a point mutation (G525R) that confers selective binding to 4-hydroxy-tamoxifen (4OHT) [25, 29]. This 4OHT-binding domain (referred to hereafter as ER), derived from codons 281 to 599 of the mouse ERα, was fused in frame to the C-terminal end of the SIRT1 coding region. The knock-in vector was electroporated into ES cells and clones of puromycin-resistant cells carrying the vector homologously recombined were identified by Southern blots of BamH1 digested genomic DNA probed with the genomic fragment indicated in Fig 1A.

RNA isolated from clones of ES cells carrying one *sirt1*^*ER*^ allele showed that the mRNA encoding SIRT1-ER was present at higher levels than the mRNA encoding the wild type SIRT1 protein (Fig 1B). The 1.8 kilobase 3’-UTR of the SIRT1 mRNA harbors the binding sites for a large number of micoRNAs [30, 31]. Many of these microRNAs have been shown to reduce the level of SIRT1 expression [32] so the removal of the 3’UTR is predicted to result in a more stable mRNA [33, 34]. Addition of 4OHT to the medium did not change the abundance of either transcript (Fig 1B).

Western blots of protein isolated from the ES cells showed the presence of the SIRT1-ER protein in cells carrying the *sirt1*^*ER*^ allele (Fig 1C). We had expected the SIRT1-ER fusion protein to be more abundant than the SIRT1 protein in the heterozygous *sirt1*^*+/ER*^ cells because the transcript encoding SIRT1-ER is more abundant and because the absence of the 3’UTR and its miRNA binding sites was expected to be more efficiently translated [35]. However, we found that the SIRT1-ER protein was present at only 25–30% of the level of the SIRT1 protein derived from the *sirt1*^*+*^ allele. In cells grown for 24 hr in the presence of 4OHT, the SIRT1-ER protein increased to levels commensurate with those of the SIRT1 protein derived from the normal *sirt1*^*+*^ allele.

**Fig 2:**
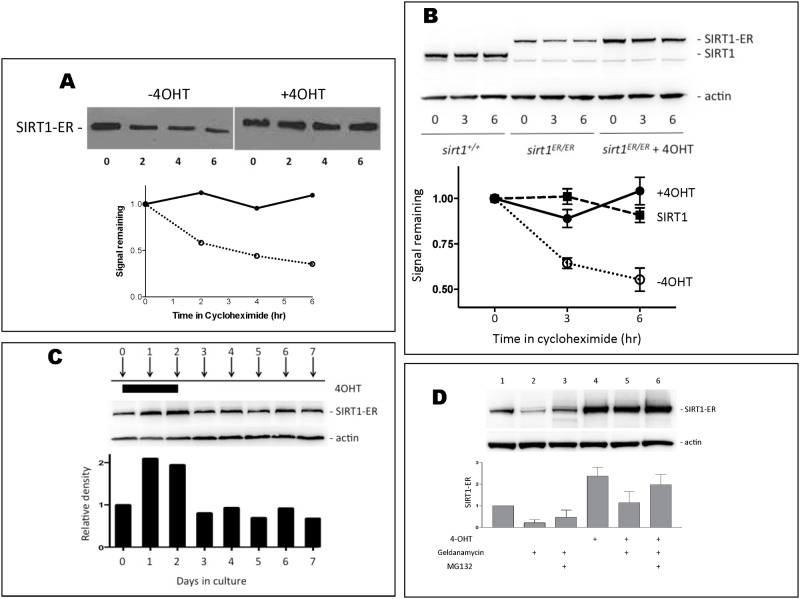
The SIRT1-ER protein is stabilized by 4OHT. A. 293T cells were transfected with a plasmid carrying the SIRT1-ER coding region driven by the CMV promoter, the cells were either treated or not with 4OHT (30 nM) and 24 hr later cycloheximide (10 μg/ml) was added to each culture. At 2 hr intervals thereafter, protein was harvested from cultures and immunoblots probed for SIRT1 and β-actin. The densitometry of the blot is shown below and indicates that the stability of SIRT1-ER is increased in the presence of 4OHT. B. MEFs derived from *sirt1*^*+/+*^ or *sirt1*^*ER/ER*^ embryos were cultured for 24 hr in the absence or presence of 100 nM 4OHT before the addition of cycloheximide to each culture. Cultures were harvested 0, 3 and 6 hr after addition of cycloheximide, protein extracted and immunoblots probed for SIRT1 and β-actin. One representative blot is shown and the densitometry from 5 independent experiments was used to create the decay curve below. C. *sirt1*^*ER/ER*^ MEFs were cultured for 2 days in the presence of 100 nM 4OHT and then the drug was removed and cells cultured for an additional 5 days. Extracts were prepared at daily intervals and immunoblots probed with antibodies to SIRT1 and β-actin. Quantitation of the SIRT1-ER/β-actin signals is shown below the blot. D. Cultures of *sirt1*^*ER/ER*^ MEFs were grown for 24 hr in 0 or 100 nM 4OHT before the addition of geldanamycin (0.5 μg/ml) or of geldanamycin plus MG132 (1 μM). Following an additional 4 hr incubation, cells were harvested and immunoblots probed for SIRT1 and for β-actin. Quantitation of the signal from the SIRT1-ER protein is shown in the bar graph from 3 independent experiments.

To investigate why the SIRT1-ER protein was less abundant than expected we measured its stability in cells. We transfected 293T cells with a plasmid encoding SIRT1-ER and after 24 hr, the cells were treated with cycloheximide. Protein was isolated at intervals thereafter to measure the rate of decay of SIRT1-ER (Fig 2A). The abundance of the SIRT1-ER protein decayed by more than 50% during the 6 hr exposure to cycloheximide. However, in cells exposed to 4OHT as well as cycloheximide the amount of SIRT1-ER did not appreciably decline suggesting that 4OHT stabilizes the fusion protein.

Mouse embryo fibroblasts (MEFs) were isolated from embryos homozygous for the *sirt1*^*ER*^ allele. These *sirt1*^*ER/ER*^ MEFs were treated with or without 4OHT for 24 hours and then exposed to cycloheximide. The SIRT1-ER synthesized from the genomic *sirt1*^*ER*^ genes was also found to be unstable in the absence but not in the presence of 4OHT (Fig 2B). In experiments on wild type MEFs, SIRT1 was stable for over 6 hr in cycloheximide. Thus, we infer that the ER domain confers instability to the SIRT1-ER fusion protein and that the 4OHT ligand reverses this destabilizing effect.

To determine if stabilization of SIRT1-ER by 4OHT is reversible, we isolated protein at daily intervals from *sirt1*^*ER/ER*^ cells exposed to 4OHT for their first 48 hr in culture. SIRT1-ER increased in abundance within 24 hr of addition of 4OHT and returned to pre-induced levels within 24 hr of removal of 4OHT from the medium (Fig 2C). Thus the abundance of SIRT1-ER is reversibly induced by 4OHT in cultured cells.

The ER domain is thought to regulate the activity of proteins to which it is fused by binding to hsp90 in a chaperone complex [36, 37]. Hsp90 bound to the ER domain is thought to maintain this domain in a configuration able to efficiently bind its ligand. We were unable to co-immunoprecipitate hsp90 with SIRT1-ER but found that after 6 hours exposure to geldanamycin, an inhibitor of hsp90, about 70% of the SIRT1-ER protein was degraded (Fig 2D). This degradation was partially prevented by the proteasome inhibitor, MG132, suggesting that SIRT1-ER is targeted for proteasomal degradation in the absence of hsp90 activity. Less geldanamycin-induced degradation of SIRT1-ER occurred in cells grown in the presence of 4OHT suggesting that ligand binding stabilizes SIRT1-ER by removing it from hsp90. Geldanamycin did not destabilize the SIRT1 protein (data not shown).

**Fig 3:**
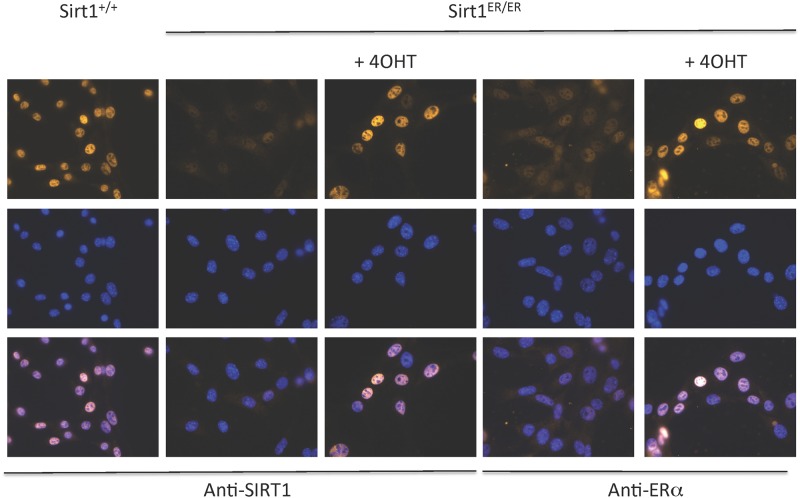
The SIRT1-ER protein is located in the nucleus in the presence and absence of 4OHT. MEFs derived from wild type (*sirt1*^*+/+*^) or homozygous mutant (*sirt1*^*ER/ER*^) embryos were cultured, treated for 24 hr with 0 or 100 nM 4OHT and fixed for immunofluorescence. Antibodies to SIRT1 react with protein in both wild type and mutant cells while antibodies to the murine estrogen receptor α (ERα) reacted with the nuclei from the *sirt1*^*ER/ER*^ cells. Cell nuclei were counterstained with DAPI.

We carried out immunofluorescence experiments on *sirt1*^*ER/ER*^ MEFs using antibodies specific for SIRT1 and for ER (Fig 3). The SIRT1-ER protein was reactive with both antibodies in *sirt1*^*ER/ER*^ MEFs. Neither antibody detected anything in MEFs from *sirt1*^*-/-*^ embryos and *sirt1*^*+/+*^ cells did not react with antibody to ER (data not shown). The SIRT1-ER protein is present exclusively in the nucleus of interphase cells as is the SIRT1 protein. The intensities of the SIRT1 and ER signals from *sirt1*^*ER/ER*^ cells increased 3–4 fold when cells were cultured for 24 hr in the presence of 4OHT, consistent with the results shown in the immunoblot of Fig 1C. The intensity of the signal from SIRT1-ER was variable from cell to cell whereas the SIRT1 signal in *sirt1*^*+/+*^ cells was more uniform.

### Catalytic activity of SIRT1-ER

**Fig 4:**
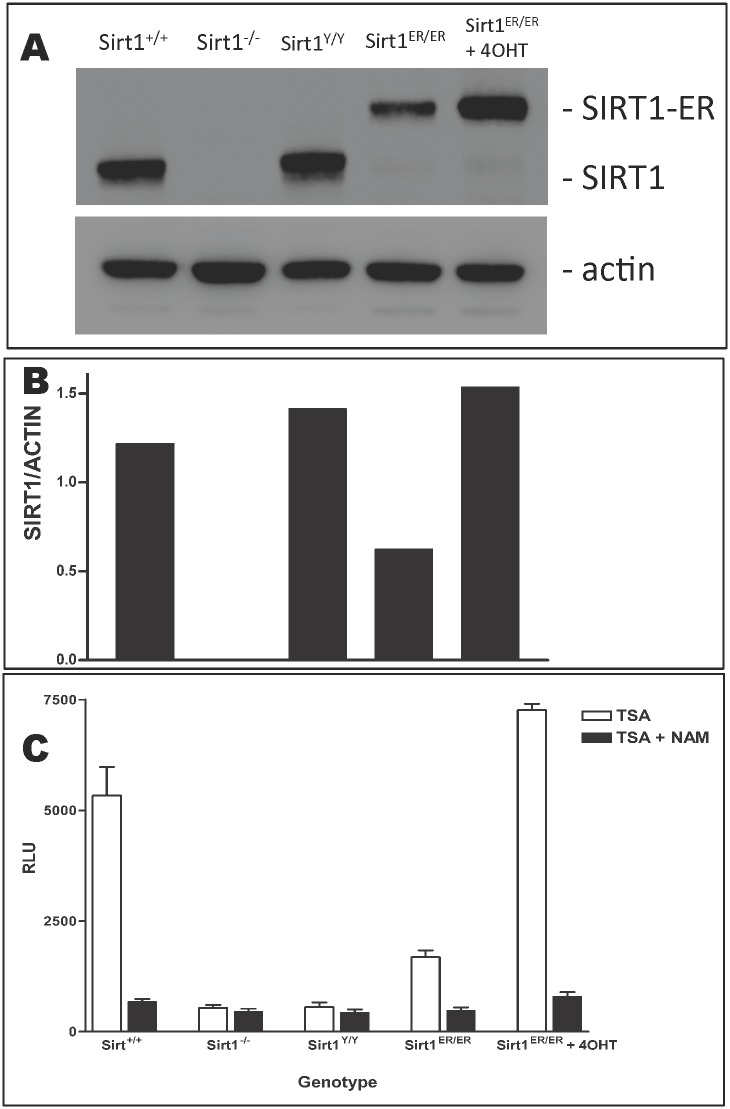
Activity of the SIRT1-ER fusion protein. A. Mouse embryo fibroblasts (MEFs) derived from embryos of the indicated *sirt1* genotypes were harvested and proteins subject to PAGE and prepared for immunoblotting with antibodies to SIRT1 and to β-actin. The cells labeled “*sirt1*^*ER/ER*^ + tam” in the right lane were treated for 24 hr with 4OHT before harvest. B. The ratio of the signals from SIRT1 and β-actin immunoblots from panel A are plotted. C. Proteins from the 5 cell cultures were immunoprecipitated with the anti-SIRT1 antibody followed by assessment of the sirtuin deacetylase activity carried out (open bars) in the presence of trichostatin A (TSA, 4 μM, an inhibitor of type 1 and 2 HDACs) or (black bars) in the presence of both TSA and nicotinamide (NAM, 10 mM, an inhibitor of sirtuins or type 3 HDACs). Standard errors are from 3 assessments of enzyme activity from independent cell cultures. Cells labeled *sirt1*^*-/-*^ and *sirt1*^*Y/Y*^ are homozygous for the null [17] and point mutations [15] of *sirt1* respectively.

To investigate the activity of SIRT1-ER derived from the *sirt1*^*ER*^ gene, we used an antibody directed to the N-terminal amino acids of SIRT1 to immunoprecipitate SIRT1-ER and SIRT1 proteins from MEFs of various genotypes and measured the sirtuin activity in the immunoprecipitates (Fig 4). The abundance of the SIRT1-ER protein in *sirt1*^*ER/ER*^ cells was 2.5-fold higher following 4OHT exposure while the enzyme activity was about 5-fold higher suggesting that the 4OHT ligand increased both the amount of SIRT1-ER protein (by 2.5 fold) and its specific activity (by an additional 2 fold). The sirtuin activity of SIRT1-ER from untreated *sirt1*^*ER/ER*^ cells was about 30% that of SIRT1 in *sirt1*^*+/+*^ cells but increased to 130% that in *sirt1*^*+/+*^ cells following 4OHT treatment.

**Fig 5:**
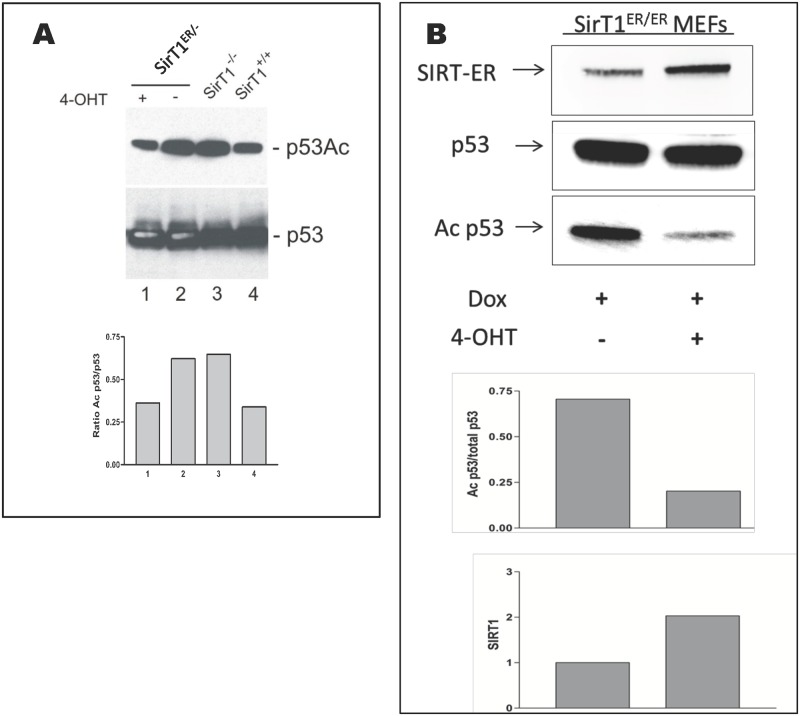
Evidence for SIRT1-ER deacetylase activity in cultured cells. A. *sirt1*^*+/ER*^ embryonic stem cells were transfected with the knockout (KO) insertion vector [17] and clones screened for those with the KO inserted into the wild type allele. A clone of these sirt1^ER/-^ cells was isolated and treated for 24 hr with 4OHT (30 nM) and then irradiated with ultraviolet radiation (30J/m^2^), incubated for 18 hr and harvested for immunoblotting. Blots were probed with antibodies reactive with acetylated-p53 (Ac-K379) and total p53 protein as indicated. ES cells that are *sirt1*^*-/-*^ and *sirt1*^*+/+*^ [60] were irradiated in parallel to show the effect on acetylated p53 of the absence and presence of SIRT1 activity respectively. The intensity of the acetyl-p53 signal is plotted on the bar graph. B. *sirt1*^*ER/ER*^ MEFs were treated or untreated for 24 hr with 4OHT before 1 μM doxorubicin was added for an additional 24 hr. Immunoblots were probed for SIRT1, acetyl-p53 and total p53 as indicated. The bar graphs show the intensity of the acetyl-p53 signal (upper graph) and of SIRT1-ER (lower graph).

One established substrate for SIRT1 deacetylase activity is p53. Antibodies reactive with p53 acetylated on lysine 379 were used to estimate the activity of SIRT1-ER in cells in culture. p53 was induced in ES cells (Fig 5A) by ultraviolet irradiation (30 J/m^2^) and in MEFs (Fig 5B) by doxorubicin (1 μM). The levels of total p53 and Ac-p53 were assessed in immunoblots. In both cell types, the Ac-p53 signal was lower in cells grown in the presence of 4OHT consistent with the induction of SIRT1-ER sirtuin activity by the ligand.

### Characteristics of *sirt1*^*ER/ER*^ mice

**Fig 6:**
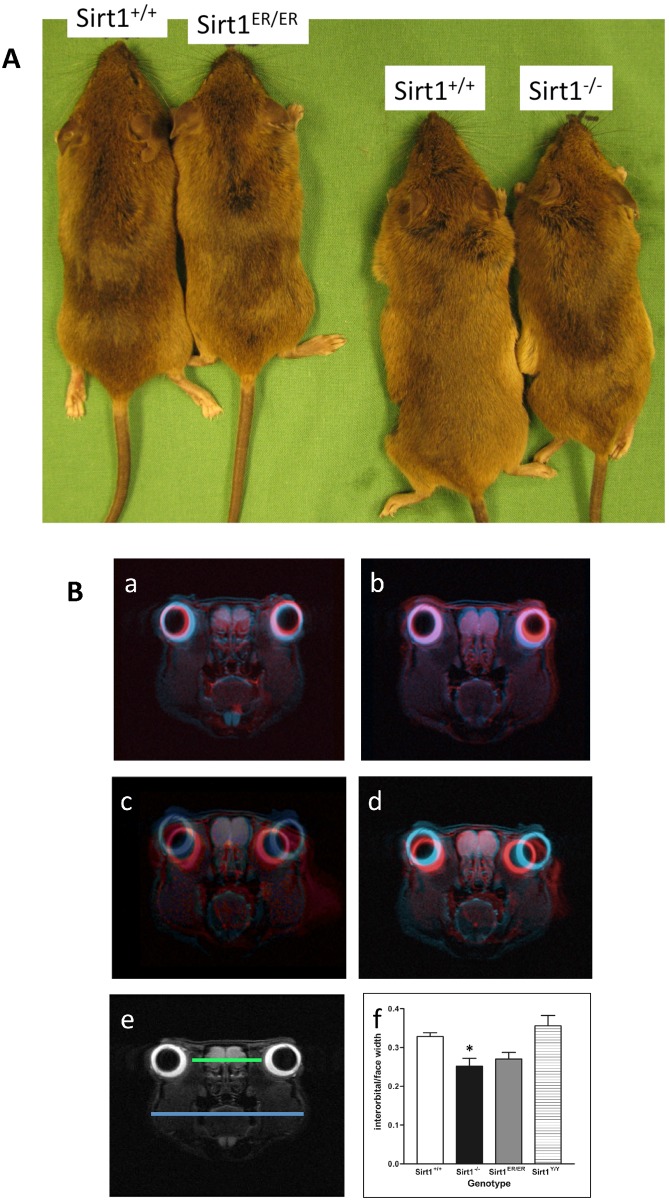
*sirt1*^*ER/ER*^ mice have craniofacial abnormalities similar to those present in *sirt1*^*-/-*^ animals. A. Photographs of *sirt1*^*+/+*^ and *sirt1*^*ER/ER*^ littermates (on the left) and *sirt1*^*+/+*^ and *sirt1*^*-/-*^ littermates (on the right). The *sirt1*^*-/-*^ and *sirt1*^*ER/ER*^ animals have a shortened snout compared to their *sirt1*^*+/+*^ littermates. *sirt1*^*-/-*^ animals are also smaller than their normal littermates. B. MRI images of the heads of animals of various genotypes show that the interorbital distance is reduced in *sirt1*^*-/-*^ and *sirt1*^*ER/ER*^ mice. The *sirt1*^*+/+*^ image is colored blue while the images from the other genotypes were rendered red before superimposition. Panel A is from *sirt1*^*Y/Y*^, B from *sirt1*^*+/-*^, C from *sirt1*^*-/-*^, and D from *sirt1*^*ER/ER*^. Quantitation of interorbital distance shown in the bottom bar graph was measured by taking the ratio of the green over the blue lines. Measurements were made from 3 MRI images of each genotype from male animals of 3–4 months of age. * indicates P<0.05.

*sirt1*^*+/ER*^ ES cells were used to establish a colony of mice carrying the *sirt1*^*ER*^ allele. Since fusion of hormone-binding domains to various proteins generally suppressed that protein activity [24] and since knock-in of ER into the p53 locus resulted in a null allele of p53 [38], we had expected that the *sirt1*^*ER/ER*^ mice would resemble *sirt1*^*-/-*^ animals [17]. This was not the case. Rather, *sirt1*^*ER/ER*^ mice have a phenotype only slightly resembling that of the *sirt1*^*-/-*^ mice. *sirt1*^*-/-*^ animals are smaller than normal and suffer high levels of perinatal lethality while *sirt1*^*ER/ER*^ mice are normal size (Fig 6A and S1 Fig) and emerge at Mendelian ratios from matings between heterozygous *sirt1*^*ER/+*^ animals (Table 1). Whereas both sexes of *sirt1*^*-/-*^ animals are sterile, female *sirt1*^*ER/ER*^ mice are fertile while males have mildly reduced fertility (Table 2). Interestingly, *sirt1*^*ER/ER*^ mice have a number of characteristic craniofacial abnormalities shared with the *sirt1*^*-/-*^ mice. These include a short snout (Fig 6A) and reduced intraorbital distance (Fig 6B). However, the disorganization of the rugae of the hard palate, a phenotype of *sirt1*^*-/-*^ mice, was not evident in the *sirt1*^*ER/ER*^ animals (S2 Fig).

**Table 1:** Genotypes from *sirt1*^*ERtm/+*^ intercrosses.

	ER/ER	ER/^+^	^+^/^+^
**Number at weaning**	**53**	**120**	**57**
**%**	**23**	**52**	**25**

**Table 2:** 4 month test mating to sirt1^+/+^.

Female ER/ER	Male ER/ER
8/9 fertile	6/10 fertile
122 pups total	121 pups total
6.8 pups/litter	7.1 pups/litter

Male and female mice with the *sirt1*^*ER/ER*^ genotype were caged with wild type animals of the opposite sex and the number and size of the litters born over the next 4 months recorded.

**Fig 7:**
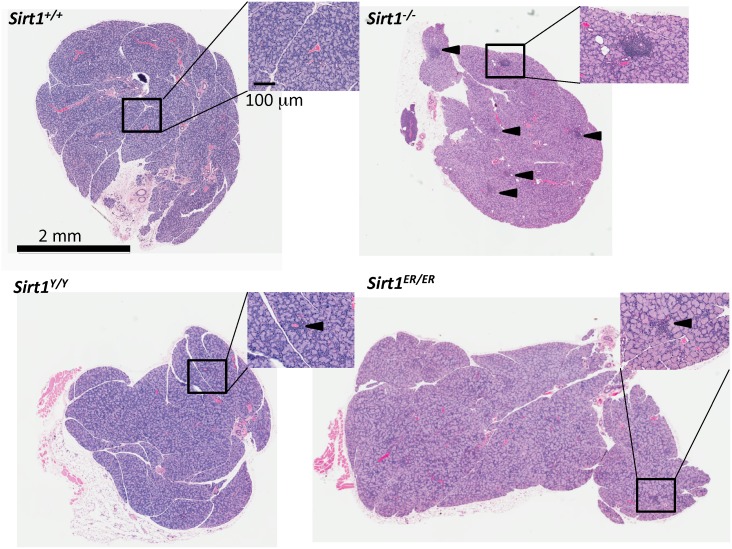
Histological sections of lacrimal glands. Extraorbital lacrimal glands from 6 month old mice of the indicated 4 genotypes were harvested, fixed, sectioned and stained with hematoxylin and eosin. The regions in boxes are magnified to show the foci of lymphocyte infiltration. Arrowheads in the *sirt1*^*-/-*^ section indicate the multiple nests of lymphoid cells found in lacrimal glands from animals of this genotype. Magnification bars; main image 2mm, inset 100 μm.

*sirt1*^*-/-*^ animals develop an autoimmune condition [39] at least in part due to a failure to elaborate a varied spectrum of Treg cells [40]. A sensitive readout of the autoimmunity conferred by SIRT1 deficiency is the development of lymphoid infiltration of lacrimal and salivary glands. Although almost all *sirt1*^*-/-*^ mice had extensive lymphoid infiltration of both glands, the *sirt1*^*ER/ER*^ animals had fewer and smaller nests of lymphoid cells in these two glands (Table 3 and Fig 7). Autoantibodies referred to as La and Ro, characteristics of various human autoimmune conditions, are elevated in *sirt1*^*-/-*^ animals but *sirt1*^*ER/ER*^ mice did not have titers of these antibodies higher than the *sirt1*^*+/+*^ controls (S3 Fig).

**Table 3:** Percentage of lacrimal and salivary glands scored for nests of lymphocytes.

	Lacrimal Gland		Salivary Gland	
	2–6 mo.	7–12 mo.	2–6 mo.	7–12 mo.
**Sirt1**^**+/+**^	**0** (0/9)	**20** (2/10)	**0** (0/9)	**20** (2/10)
**Sirt1**^**-/-**^	**86** (12/14)	**100** (5/5)	**43** (6/14)	**80** (4/5)
**Sirt1**^**Y/Y**^	**89** (8/9)	**69** (11/16)	**33** (3/9)	**93** (15/16)
**Sirt1**^**ER/ER**^	**0** (0/6)	**61** (11/18)	**17** (1/6)	**83** (15/18)

Most of the characteristics of *sirt1*^*ER/ER*^ mice resemble *sirt1*^*+/+*^ rather than *sirt1*^*-/-*^ animals (Table 4). Thus, in the absence of 4OHT, the *sirt1*^*ER/ER*^ mice have a phenotype resembling that expected of a mild hypomorph rather than a null. This result is consistent with the fact that the sirtuin activity of SIRT1-ER in *sirt1*^*ER/ER*^ is about 30% that of SIRT1 in *sirt1*^*+/+*^ cells.

**Table 4:** Characteristics of mice bearing induced mutations of the *sirt1* gene.

	SirT1^-/-^	SirT1^Y/Y^	SirT1^ER/ER^
**Craniofacial features**	**+**	**+**	**+**
**Male infertility**	**+**	**+**	**+/-**
**Lacrimal gland infiltration**	**+**	**+/-**	**+/-**
**Perinatal lethality**	**+**	**+**	**-**
**Elevated respiration**	**+**	**+**	**-**
**Small size**	**+**	**+**	**-**
**Eyelid defect**	**+**	**+/-**	**-**
**Lethargy**	**+**	**-**	**-**
**Female infertility**	**+**	**-**	**-**
**Lethal on inbred background**	**+**	**-**	**-**

**Fig 8:**
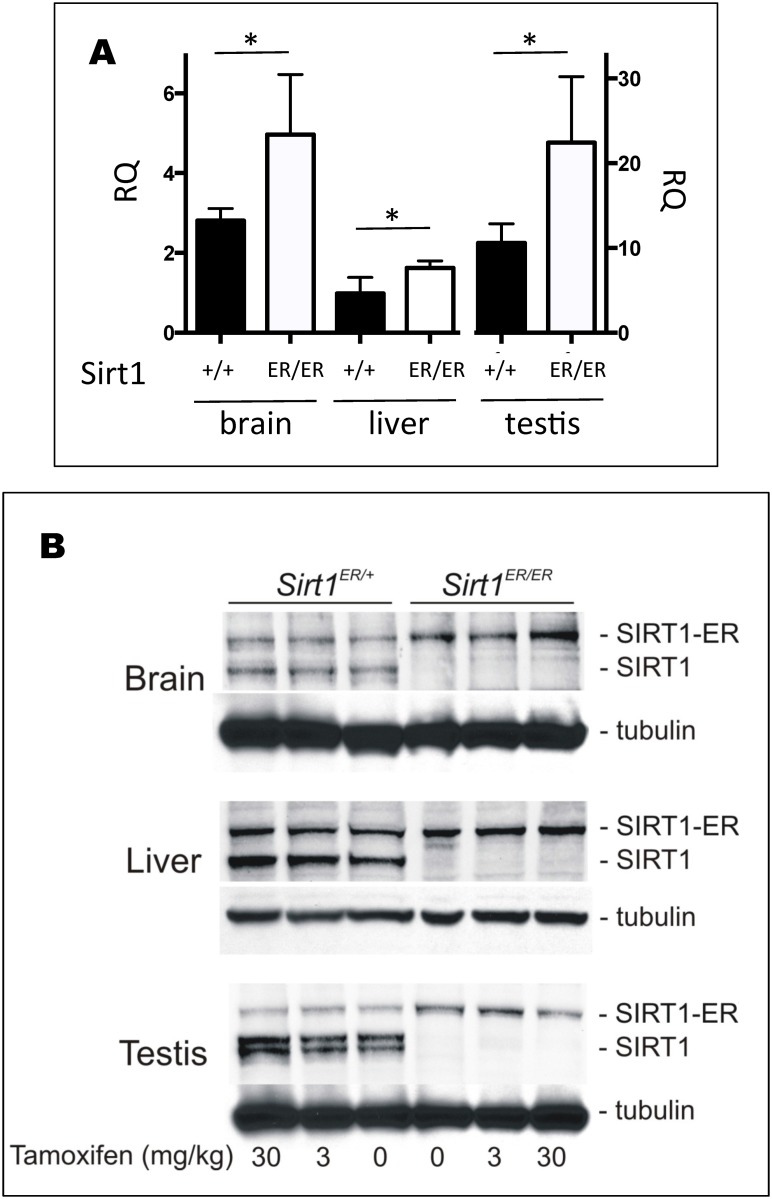
SIRT1-ER mRNA and protein in *sirt1*^*+/ER*^ and *sirt1*^*ER/ER*^ tissues. A. Levels of mRNA from the *sirt1*^*ER*^ gene are higher than those from *sirt1*^*+*^. RNA was isolated from brain, liver and testes of *sirt1*^*ER/ER*^ and *sirt1*^*+/+*^ mice and used for quantitative assessment of SIRT1 mRNA levels by comparative PCR. RNA levels varied between different tissues but the mRNA from *sirt1*^*ER/ER*^ animals was 50–100% higher than that from *sirt1*^*+/+*^ mice. Results are from 3 animals of each genotype. * indicates p<0.05. B. Levels of SIRT1 and SIRT1-ER in mice exposed for 1 week to low doses of tamoxifen. Brain, liver and testes of *sirt1*^*+/ER*^ and *sirt*^*ER/ER*^ mice were harvested from animals fed diets containing the indicated concentrations of tamoxifen for 1 week. Immunoblots were probed with antibodies against SIRT1 and α-tubulin.

Quantitative PCR indicated that the levels of the SIRT1-ER mRNA are about 2 fold higher than those of the SIRT1 mRNA in the same tissues (Fig 8A), a result consistent with the cell culture results (Fig 1B) and the expectation that the lengthy 3’UTR of the SIRT1 transcript is the target for a number of miRNAs that target its mRNA for destruction. However, direct measurement of SIRT1-ER mRNA stability in *sirt1*^*ER/ER*^ MEFs indicated that its half-life of 3 hrs is similar to that of SIRT1 mRNA (S4 Fig) so the reason for the higher levels of SIRT1-ER mRNA remains unclear.

The abundance of SIRT1-ER protein in the testis was lower than SIRT1 (Fig 8B) as we observed in cell cultures; however, in brain and liver, the SIRT1-ER protein was present at levels similar to those of SIRT1 suggesting that the protein instability conferred by the ER varies between tissues and is perhaps related to the tissue distribution of hsp90. Low concentrations of tamoxifen in the diet resulted in no increase in SIRT1-ER abundance.

**Fig 9:**
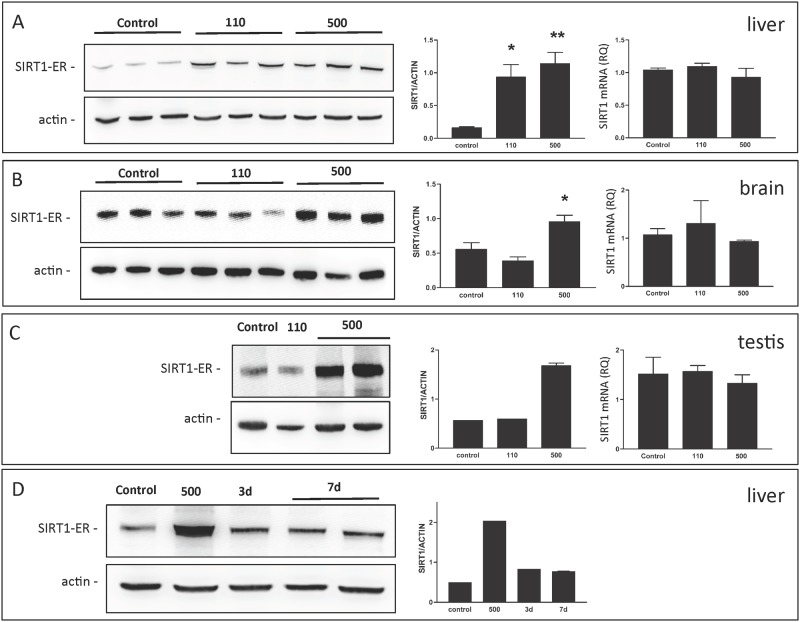
Reversible induction of SIRT1-ER *in vivo*. Panels A, B and C are analyses of liver, brain and testes from *sirt*^*ER/ER*^ mice fed a diet supplemented with 0, 110, or 500 mg/kg tamoxifen immediately following weaning at 3 weeks of age. After 6 months tissues were harvested and immunoblots probed with antibodies to SIRT1 and to β-actin. The immunoblots are shown on the left; the ratio of the SIRT1-ER/β-actin signal is in the bar graph to the right of the blot. Comparative PCR of the SIRT1-ER mRNA is shown on the right of each panel. Significant differences from controls (*, p <.05; **, p, <.01). D. Liver was harvested from mice fed the 500 mg/kg tamoxifen diet for 6 months before being fed normal chow for 3 or 7 days. The immunoblot was probed with antibody to SIRT1 and β-actin.

Very high concentrations of tamoxifen in the diet are normally required to induce activation of fusion protein activity *in vivo* [41]. We found that *sirt1*^*ER/ER*^ mice developed and maintained elevated levels of SIRT1-ER protein when fed diets containing tamoxifen at 110 and 500 mg/kg (Fig 9). The induction of SIRT1-ER was dose dependent and not accompanied by a change in mRNA level, consistent with the idea that the increased abundance of SIRT1-ER is due to stabilization of the protein and/or its increased rate of translation. There were some differences in the response of different tissues to tamoxifen feeding perhaps reflecting drug distribution and metabolism.

**Fig 10:**
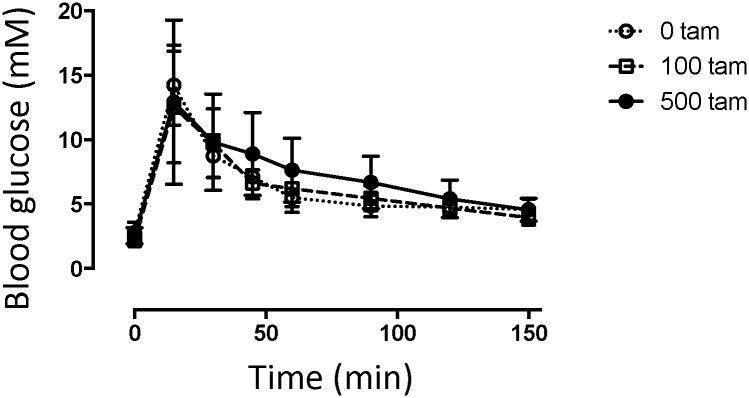
Oral glucose tolerance tests on mice maintained on tamoxifen for 6 months. Male *sirt1*^*ER/ER*^ mice were fed diets containing 0, 110, or 500 mg/kg tamoxifen for 6 months. Each animal was then given oral glucose tolerance tests twice separated by 1 week. N = 4 mice on the 500 mg/kg dose and n = 3 for 0 and 110 mg/kg tamoxifen.

*sirt1*^*ER/ER*^ mice were transferred to diets containing 110 and 500 mg/kg tamoxifen starting at 3 weeks of age and were maintained for up to 12 months these diets. As expected from the anti-estrogenic effects of the drug, tamoxifen caused both sexes to be sterile but the animals otherwise seemed to tolerate the diets well. Oral glucose tolerance tests on *sirt1*^*ER/ER*^ mice maintained for 6 months on tamoxifen indicated only a slight positive effect of the elevated SIRT1-ER (Fig 10). This result is consistent with the findings of Boutant et al [42] who also found that overexpression of SIRT1 using transgenesis did not significantly improve glucose metabolism and insulin sensitivity in mice fed normal chow diets.

Transgenic mice expressing SIRT1 at levels 2–3 fold higher than normal have been reported to be protected from developing symptoms of metabolic syndrome when animals are fed a high fat diet [43]. Our attempts to carry out this kind of study were frustrated because at the doses of tamoxifen required for induced SIRT1-ER expression, the drug has other physiological effects. In particular, tamoxifen is known to induce destruction of body fat [44] so experiments aimed at assessing the metabolic effects of elevated SIRT1-ER activity are confounded by the effects of tamoxifen itself. Indeed, we found that *sirt1*^*ER/ER*^ fed a high fat diet developed much reduced fat depots when the chow contained tamoxifen; however, similar effects were seen in wild type mice so the reduced fat accumulation in the *sirt1*^*ER/ER*^ was likely a result of the effects of tamoxifen on fat metabolism rather than on the induction of SIRT1-ER (data not shown).

## Discussion

Elevated levels of the SIRT1 protein are thought to result in elongated lifespan along with reduced susceptibility to a wide variety of disease states including neurodegeneration, cancer, metabolic syndrome and autoimmune disease [45–48]. Establishing that SIRT1 has a causal role in forestalling disease onset is difficult because many experiments rely on the use of drugs of indeterminate specificity to increase or decrease SIRT1 activity. We report here a mouse strain in which the level of SIRT1-ER protein and activity can be reversibly modulated by 2 to 5 fold respectively. Mice carrying inducible genes using similar methodology have been very valuable in establishing the roles of tumor suppressor genes and oncogenes in the development of cancers [22].

When fused to a variety of other proteins, the ER has been shown to inactivate the host protein function whether that protein is an enzyme, such as cre [49] or caspase [50], or a transcription factor such as c-myc [29], p53 [38], E2A [51], or c-myb [52]. That SIRT1-ER appears to retain most of the catalytic activity and biological functions of SIRT1 suggests that the ER in the context of SIRT1 does not exert a strong cis-dominant repressive effect. SIRT1 is not unique in this regard as cre-ER has been reported to have residual constitutive activity when expressed at high level [53, 54]. Various other enzymes are also known to retain activity following fusion to hormone-binding domains [24].

The means by which ER effects inactivation of its protein fusion partner is not known. The ER is thought to bind to a complex of chaperones including hsp90 [36] [37] perhaps becoming sequestered into a compartment of the cell remote from the target substrate(s). Our immunofluorescence experiments indicated that SIRT1-ER (like SIRT1) resides exclusively in the nucleoplasm (except during mitosis) and that this distribution was not altered in the presence of 4OHT (Fig 3). The widely used cre-ER protein behaves differently from SIRT1-ER. Cre-ER has been reported to be broadly distributed in the cell and to become concentrated in the nuclei following the addition of tamoxifen [55]. No increase in cre-ER protein was reported following addition of ligand. However, other ER fusion proteins such as Mek1 [56] and STAT5 [57] have been reported to increase in abundance following exposure to 4OHT.

The SIRT1-ER protein might escape suppression if the rate-limiting event in SIRT1 catalysis is binding of NAD^+^, a small molecule that readily diffuses and may not be influenced by sequestering of the SIRT-ER molecule into hsp90-bound cellular sub-compartments. Another possibility is that the association of the ER with the hsp90 chaperone causes local denaturation of the fusion protein compromising critical enzymatic or binding domains. If this were the case, the catalytic sirtuin domain of SIRT1-ER might escape denaturation because the C terminal region of SIRT1 is intrinsically unstructured so the repressive ER domain may be insulated from the SIRT1 catalytic domain in the SIRT1-ER protein.

By removing the 1.8 kilobase 3’-UTR from the SIRT1-ER mRNA we removed the binding sites for a wide variety of microRNAs [30] as well as binding sites for the proteins HuR and hnRNP A1 [58, 59]. Both HuR and hnRNP A1 are reported to stabilize the SIRT1 mRNA while the miRNAs destabilize the transcript. We found that the SIRT1-ER mRNA was about 2-fold more abundant than the normal SIRT1 mRNA in both cells in culture and in tissues (Figs 1B and 7B) although the SIRT1-ER and SIRT1 mRNAs had similar rates of degradation (S4 Fig). Because it lacked the 3’ UTR binding sites for miRNAs we expected that the SIRT1-ER protein would also be translated more efficiently from its mRNA; however, we found that the SIRT1-ER protein is unstable in the absence of 4OHT and is thus generally less abundant than the SIRT1 protein. No such destabilization occurred when the enhanced green fluorescent protein (eGFP) was fused to the C terminus of SIRT1 (our unpublished results). The destabilization of the SIRT1-ER protein was exacerbated in the presence of the hsp90 inhibitor, geldanamycin, suggesting that hsp90 may be mediating the turnover of this fusion protein. Consistent with this idea is the fact that 4OHT stabilizes SIRT1-ER likely by dissociating this protein from the hsp90 complex.

Table 4 summarizes many of the characteristics of the 3 strains of mice we have created that carry mutant *sirt1* alleles. *sirt1*^*ER/ER*^ mice do not resemble the *sirt1*^*-/-*^ in most of their properties suggesting that the *sirt1*^*ER*^ allele is a hypomorph. This inference is consistent with the reduced levels of SIRT1-ER protein and activity found in cultured cells and in some tissues. Mice carrying the SIRT1(H355Y) point mutation have no detectable SIRT1 catalytic activity but the *sirt1*^*Y/Y*^ mice are also less severely affected than those homozygous for the null allele, *sirt1*^*-/-*^, suggesting that some of the functions of the SIRT1 protein do not require its deacetylase activity [15].

Many of the biological processes affected by SIRT1 have been established by using siRNAs to knockdown SIRT1 protein levels. Often a knockdown in the 25–50% range is sufficient to create a phenotype both *in vivo* and *in vitro*. This range of SIRT1-ER modulation is achieved with 4OHT so we expect that if SIRT1 activity were responsible for biological effects, we should be able to see biological events meaningfully modulated in *sirt1*^*ER/ER*^ cells or tissues treated with 4OHT. Although tamoxifen and 4OHT are not innocuous, the capability of modulating SIRT1-ER activity with these drugs adds an additional means of assessing the role of SIRT1 in physiological processes.

## Supporting information

S1 Fig *sirt1*^*ER/ER*^ mice are similar in size to their *sirt1*^*+/+*^ littermates. *sirt1*^*+/ER*^ animals were mated and their *sirt1*^*+/+*^, *sirt1*^*+/ER*^, and *sirt1*^*ER/ER*^ offspring were weighed at monthly intervals for their first 6 months (panel A). This data is from between 4 and 16 animals per genotype and sex. At 2 months after weaning the weights of between 10 and 31 animals were compared in panel B.
(TIF)

S2 Fig The hard palate rugae are disorganized in *sirt1*^*-/-*^ mice. Upon necropsy at 3 months of age, the palate of 3 mice of each genotype were inspected and photographed. Images were uploaded to Powerpoint and the drawing tool was used to trace the palatal rugae in the individual photographs. The rugae patterning was assessed for the presence of fusions, breaks, asymmetry, shortening and elongation.
(TIF)

S3 Fig *sirt1*^*-/-*^ mice develop La and Ro autoantibodies. Serum was extracted from blood samples obtained at necropsy from mice ages 2–12 months. Levels of the autoantibodies A) La (+/+ n = 16, -/- n = 15, Y/Y n = 20, ER/ER n = 12) and B) Ro (+/+ n = 23, -/- n = 14, Y/Y n = 23, ER/ER n = 12) were measured using ELISA (Signosis, Santa Clara, USA). ***P<0.001.
(TIF)

S4 Fig Stability of SIRT1 mRNA in cells of various genotypes. Growing cultures of MEFs were treated for 3 and 6 hr with actinomycin D (5 μg/ml) and RNA from these cells isolated and used to measure the SIRT1 mRNA using primers that amplify across exons 2 and 3 of the SIRT1 mRNA.
(TIF)
