# Supplementary figures and images for: Reversible modulation of SIRT1 activity in a mouse strain
Source: PLoS One. 2017 Mar 8;12(3):e0173002. doi: 10.1371/journal.pone.0173002 (PMC5342236; doi:10.1371/journal.pone.0173002)

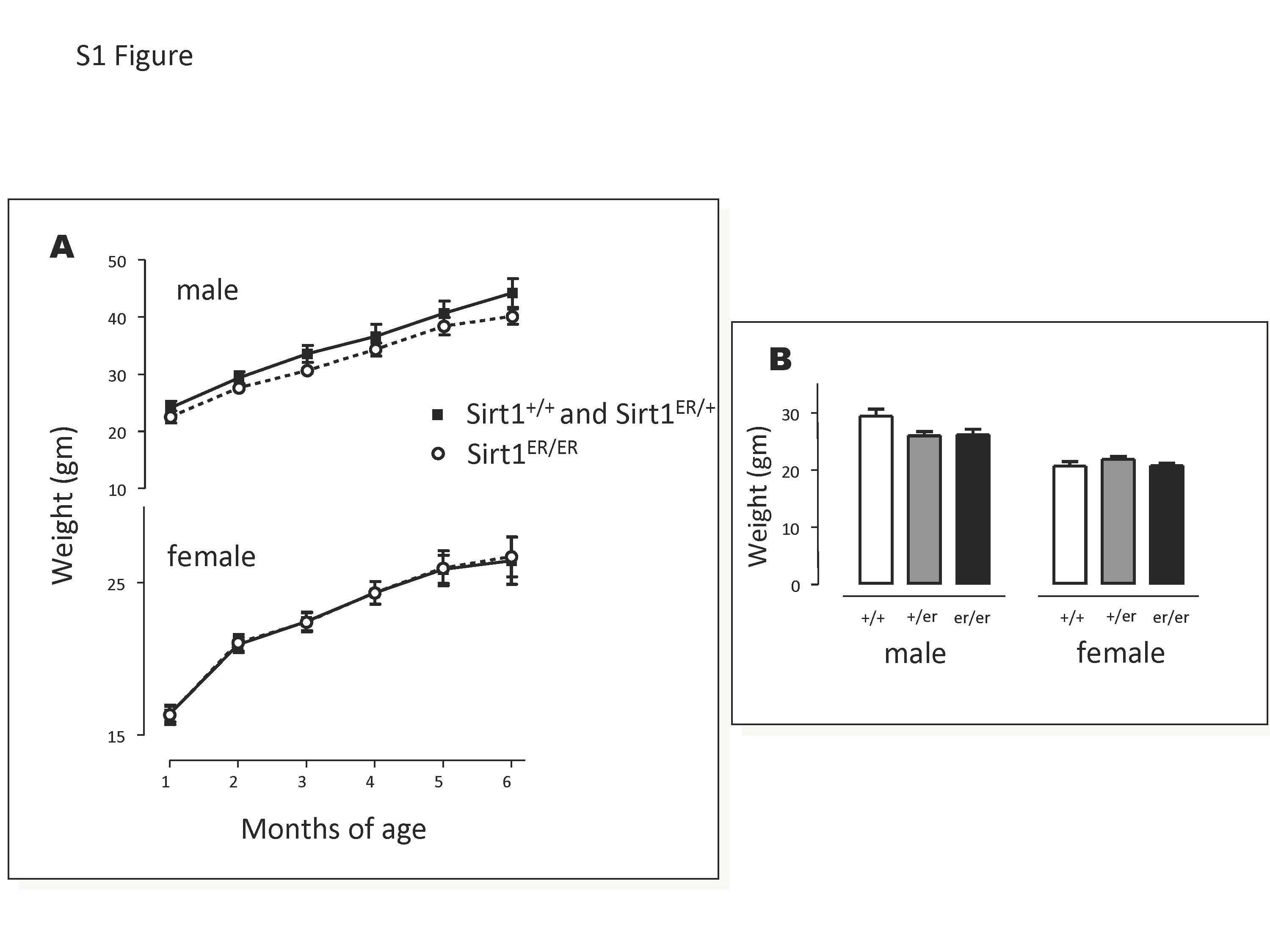

Supplement: S1 Fig — sirt1+/ER animals were mated and their sirt1+/+, sirt1+/ER, and sirt1ER/ER offspring were weighed at monthly intervals for their first 6 months (panel A). This data is from between 4 and 16 animals per genotype and sex. At 2 months after weaning the weights of between 10 and 31 animals were compared in panel B. (TIF) [file pone.0173002.s001.tif]

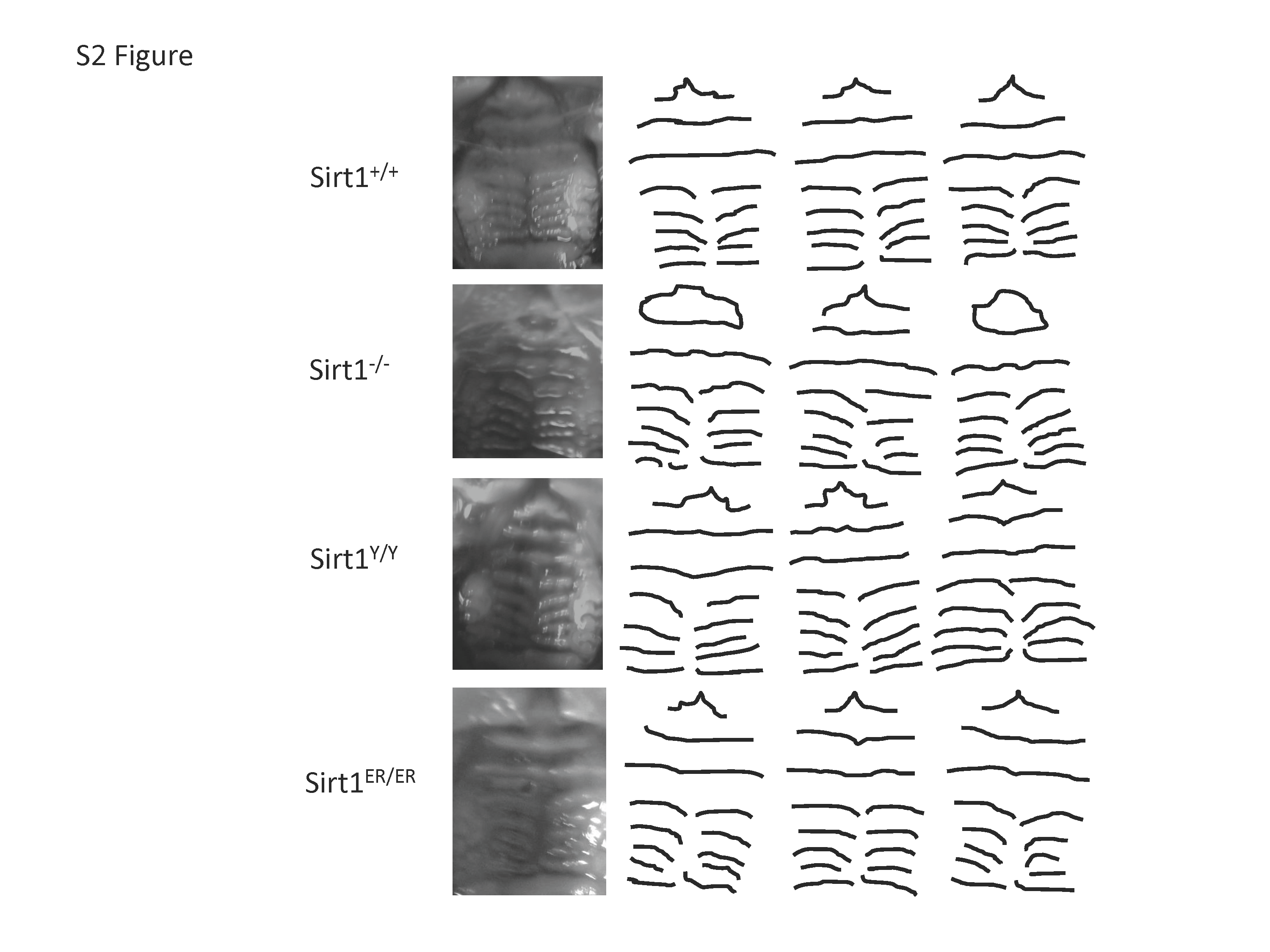

Supplement: S2 Fig — Upon necropsy at 3 months of age, the palate of 3 mice of each genotype were inspected and photographed. Images were uploaded to Powerpoint and the drawing tool was used to trace the palatal rugae in the individual photographs. The rugae patterning was assessed for the presence of fusions, breaks, asymmetry, shortening and elongation. (TIF) [file pone.0173002.s002.tif]

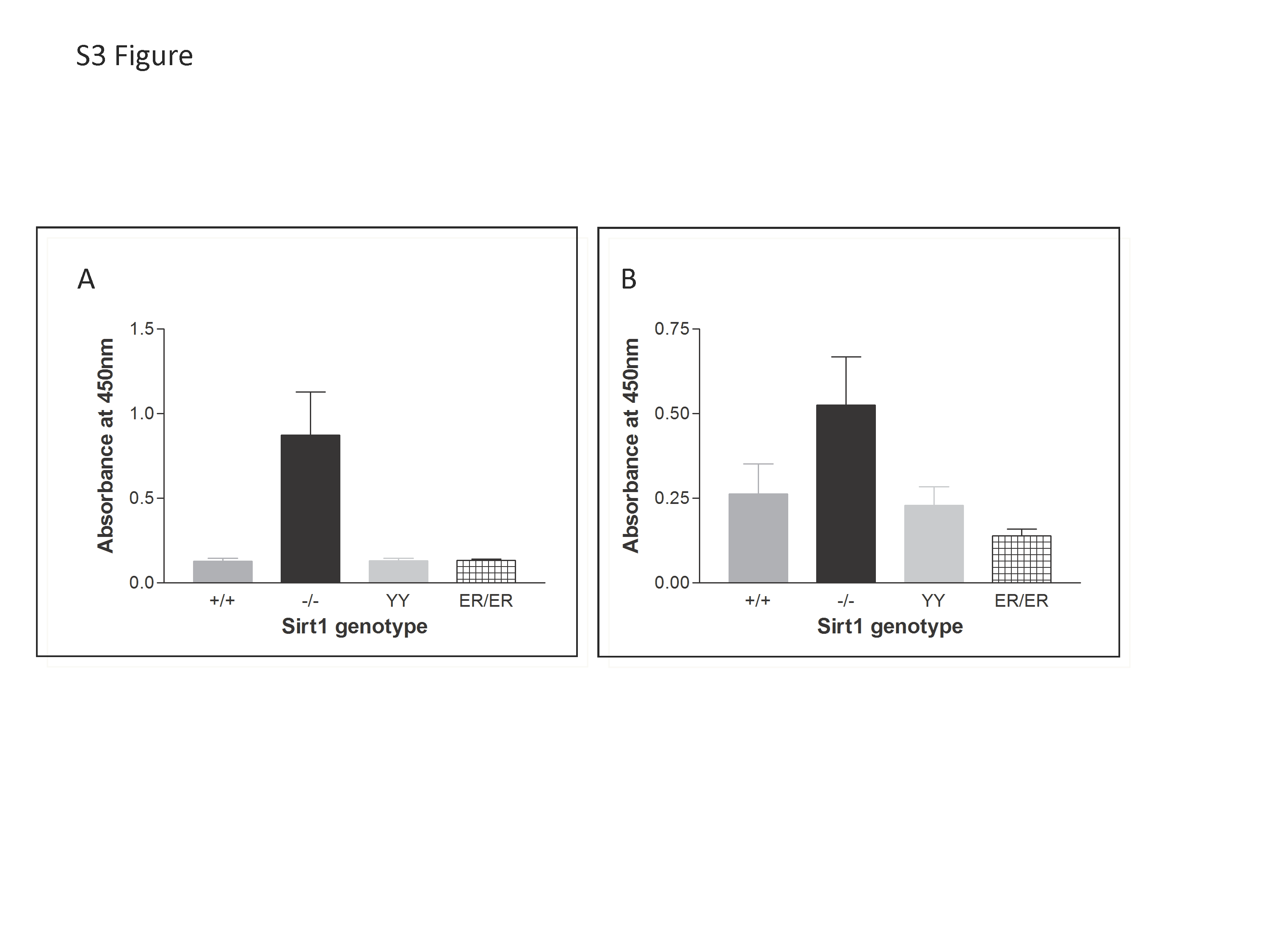

Supplement: S3 Fig — Serum was extracted from blood samples obtained at necropsy from mice ages 2–12 months. Levels of the autoantibodies A) La (+/+ n = 16, -/- n = 15, Y/Y n = 20, ER/ER n = 12) and B) Ro (+/+ n = 23, -/- n = 14, Y/Y n = 23, ER/ER n = 12) were measured using ELISA (Signosis, Santa Clara, USA). ***P<0.001. (TIF) [file pone.0173002.s003.tif]

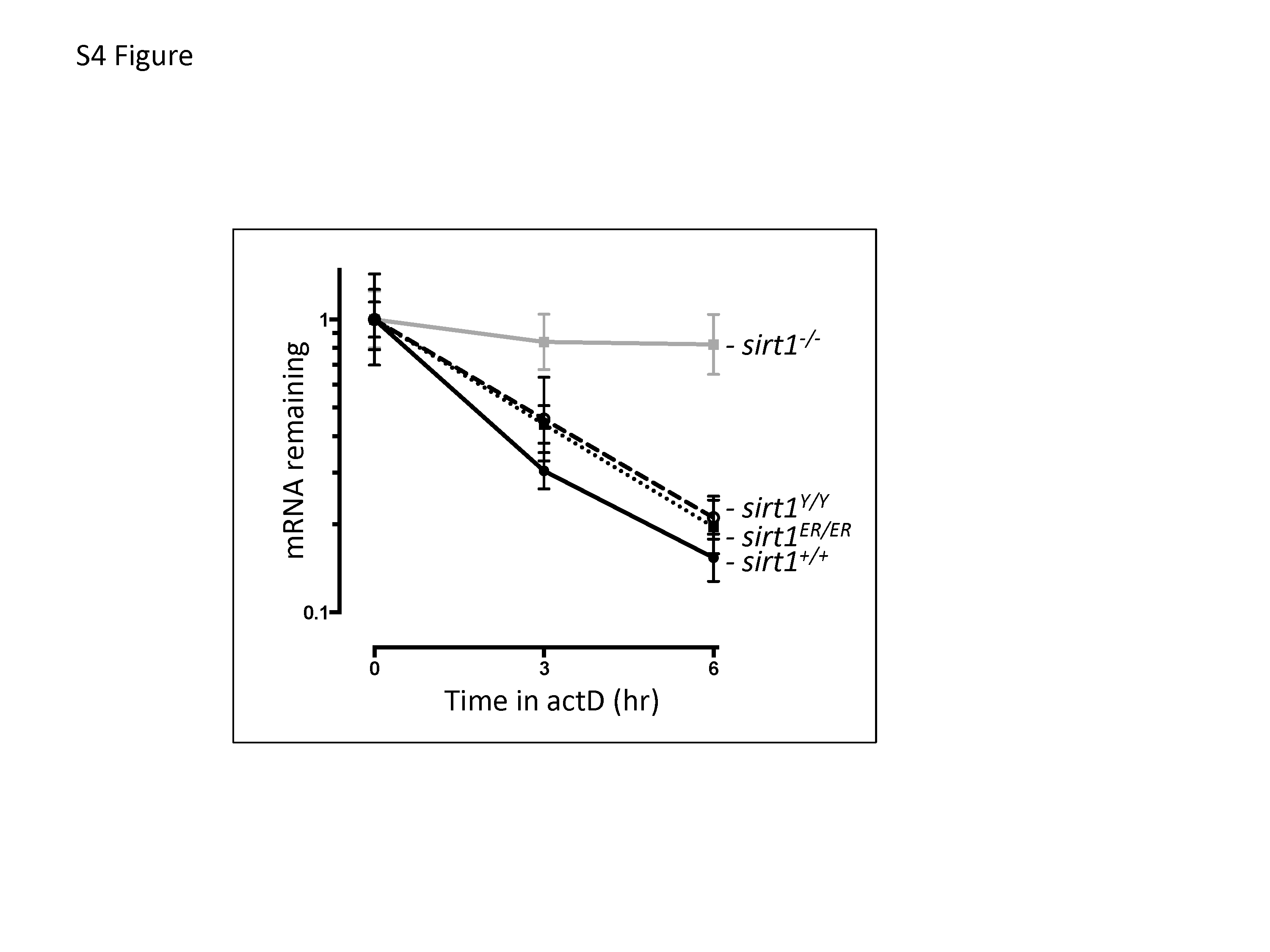

Supplement: S4 Fig — Growing cultures of MEFs were treated for 3 and 6 hr with actinomycin D (5 μg/ml) and RNA from these cells isolated and used to measure the SIRT1 mRNA using primers that amplify across exons 2 and 3 of the SIRT1 mRNA. (TIF) [file pone.0173002.s004.tif]
